# Relationship of Effective Circulating Volume with Sublingual Red Blood Cell Velocity and Microvessel Pressure Difference: A Clinical Investigation and Computational Fluid Dynamics Modeling

**DOI:** 10.3390/jcm11164885

**Published:** 2022-08-20

**Authors:** Athanasios Chalkias, Michalis Xenos

**Affiliations:** 1 Department of Anesthesiology, Faculty of Medicine, University of Thessaly, 41500 Larisa, Greece; 2 Outcomes Research Consortium, Cleveland, OH 44195, USA; 3 Committee on Shock, Hellenic Society of Cardiopulmonary Resuscitation, 10434 Athens, Greece; 4 Section of Applied and Computational Mathematics, Department of Mathematics, University of Ioannina, 45110 Ioannina, Greece

**Keywords:** cardiovascular dynamics, hemodynamics, microcirculation, anesthesia, physiology, red blood cell velocity, tissue perfusion, hemodynamic coherence, oxygen transport

## Abstract

The characteristics of physiologic hemodynamic coherence are not well-investigated. We examined the physiological relationship between circulating blood volume, sublingual microcirculatory perfusion, and tissue oxygenation in anesthetized individuals with steady-state physiology. We assessed the correlation of mean circulatory filling pressure analogue (Pmca) with sublingual microcirculatory perfusion and red blood cell (RBC) velocity using SDF+ imaging and a modified optical flow-based algorithm. We also reconstructed the 2D microvessels and applied computational fluid dynamics (CFD) to evaluate the correlation of Pmca and RBC velocity with the obtained pressure and velocity fields in microvessels from CFD (pressure difference, (Δp)). Twenty adults with a median age of 39.5 years (IQR 35.5–44.5) were included in the study. Sublingual velocity distributions were similar and followed a log-normal distribution. A constant Pmca value of 14 mmHg was observed in all individuals with sublingual RBC velocity 6–24 μm s^−1^, while a Pmca < 14 mmHg was observed in those with RBC velocity > 24 μm s^−1^. When Pmca ranged between 11 mmHg and 15 mmHg, Δp fluctuated between 0.02 Pa and 0.1 Pa. In conclusion, the intact regulatory mechanisms maintain a physiological coupling between systemic hemodynamics, sublingual microcirculatory perfusion, and tissue oxygenation when Pmca is 14 mmHg.

## 1. Introduction

Physiological hemodynamic coherence is the condition in which the systemic hemodynamic variables are translated into effective microcirculatory perfusion and oxygen delivery to the parenchymal cells [1,2]. This requires normal physiology and intact regulatory mechanisms to modulate oxygen transport to tissue. Although hemodynamic coherence was first described in 1850, only recently has it been studied in critically ill patients. Its characteristics in steady-state are not well-investigated, and no systemic variable has been consistently correlated with physiological hemodynamic coherence.

Adequate perfusion of microvascular networks is a prerequisite for tissue oxygenation [3,4,5]. Perfusion is important because hypoxia does not equate to a specific oxygen concentration; for example, many tissues function physiologically at levels equivalent to an atmosphere of 5% oxygen, and some at levels as low as 1% oxygen [6,7]. In addition, capillary rarefaction, e.g., in individuals with chronic hypertension, may jeopardize tissue oxygenation [8], but the impact of microcirculatory perfusion on the arterial load is rather limited [9]. Thus, complex mechanisms of hemodynamic coherence are required to maintain homeostasis over a very wide range of oxygen concentrations and/or perfusion changes.

Furthermore, systemic microcirculatory flow decreases with hypocapnia and increases proportionally to arterial partial pressure of carbon dioxide [10]. Other elegant studies have shown that hypoxia and hypercapnia increase mean circulatory filling pressure (Pmcf) and vascular capacitance [11,12], enhancing microcirculatory flow through a physiological adaptation to match oxygen delivery to demand [13,14]. These data could explain, in part, the vessel cluster synchronization in the microcirculatory blood flow of some organs, which changes depending on the condition of the vascular network and the blood pressure (hemodynamic coupling) [15], suggesting an interplay between effective circulating volume and tissue perfusion [11,13,14,16,17,18].

Indeed, the assessment of Pmcf is a basic parameter of functional hemodynamic monitoring. Mean circulatory filling pressure is a quantitative index of intravascular blood volume and is modifiable by vascular tone [19,20]. The Pmcf, which equals overall intravascular pressure under zero flow conditions, is an upstream pressure for venous return [20]. The latter serves as a capacitance to maintain effective circulating volume, filling of the heart, and cardiac output (CO). Based on a Guytonian model of the systemic circulation, an analogue of Pmcf (Pmca) can be derived that adequately follows intravascular volume status [21,22], its measurements are automatic, and can characterize the hemodynamic response to treatment modalities [20,23].

Decreases in Pmcf and effective circulating volume could affect microvascular red blood cell (RBC) velocity, resulting in insufficient oxygen extraction ratio (O_2_ER) [16,24,25]. On the other hand, fluid or vasopressor administration may increase stressed volume (Pmcf), eventually increasing CO and changing RBC velocity [18,26,27]. Considering that CO is determined by venous return, we hypothesized that the effective circulating volume, and thus Pmca, is associated with microcirculatory perfusion and tissue oxygenation. If this hypothesis proves to be correct, it could lead to development of an integrative monitoring method for assessing hemodynamic coherence and performance.

In the present study, we investigated the relationship between Pmca, sublingual microcirculatory perfusion, and tissue oxygenation in anesthetized individuals with steady-state physiology. In addition, computational fluid dynamics (CFD) models were developed using clinical data to evaluate the velocity and pressure fields in microvessels.

## 2. Materials and Methods

### 2.1. Design

This explorative investigation included individuals who were excluded from a previous prospective observational study due to post-enrollment use of anti-inflammatory medication. The underlying study was conducted in accordance with Good Clinical Practice guidelines, the principles of the Declaration of Helsinki, and relevant regulatory requirements. The original study was registered in ClinicalTrials.gov (NCT03851965, 22 February 2019) [28]. The Institutional Review Board of the University Hospital of Larisa approved the study (IRB no. 60580, 11 December 2018), and we obtained written individual informed consent from each participant or next-of-kin. This work is reported according to STROCSS criteria [29].

### 2.2. Study Objectives

The goals of the present study were: (1) to characterize the relationship of Pmca with sublingual microcirculation variables and RBC velocity; (2) to characterize the relationship between sublingual RBC velocity and O_2_ER; and (3) to develop CFD models using clinical data to evaluate the velocity and pressure fields in microvessels.

### 2.3. Patient Eligibility

We considered adults fulfilling the following criteria: sinus rhythm in electrocardiogram; no evidence of structural heart disease confirmed by preoperative echocardiography; and American Society of Anesthesiologists’ (ASA) physical status I.

### 2.4. Clinical Management

Before anesthesia induction, all patients received 5 mL kg^−1^ of a balanced crystalloid solution to compensate for preoperative fasting and vasodilation associated with general anesthesia. Anesthesia was induced in the supine position and included midazolam 0.15–0.35 mg kg^−1^, fentanyl 1 μg kg^−1^, ketamine 0.2 mg kg^−1^, propofol 1.5–2 mg kg^−1^, rocuronium 0.6 mg kg^−1^, and a fraction of inspired oxygen of 0.7. After tracheal intubation, patients were mechanically ventilated using a lung-protective strategy with tidal volume of 7 mL kg^−1^, positive end-expiratory pressure of 6–8 cmH_2_O, and plateau pressure < 30 cmH_2_O (Draeger Perseus A500; Drägerwerk AG & Co., Lübeck, Germany).

General anesthesia was maintained by inhalation of desflurane at an initial 1.0 minimal alveolar concentration. Thereafter, depth of anesthesia was adjusted to maintain Bispectral Index (BIS, Covidien, France) between 40 and 60 [30,31,32]. Intraoperative fraction of inspired oxygen was then adjusted to maintain an arterial oxygen partial pressure of 80–100 mmHg and normocapnia was maintained by adjusting the respiratory rate as needed [33,34,35]. Normothermia (37 °C) and normoglycemia were maintained during the perioperative period.

The radial artery was cannulated and connected to a FloTrac/EV1000 clinical platform (Edwards Life Sciences, Irvine, CA, USA) to directly measure mean arterial pressure (MAP), CO and cardiac index (CI), stroke volume (SV), stroke volume variation (SVV), and systemic vascular resistance (SVR). The internal jugular vein was cannulated with a triple-lumen central venous catheter that was connected to a pressure transducer to measure central venous pressure (CVP). Before making measurements, we confirmed that transducers were correctly leveled and zeroed, while the system’s dynamic response was confirmed with fast-flush tests. Artifacts were detected and removed when documented as such and when measurements were out-of-range or systolic and diastolic pressures were similar or abruptly changed (≥40 mmHg decrease or increase within 2 min before and after measurement). Oxygen extraction ratio was calculated as the ratio of oxygen consumption (VO_2_) to oxygen delivery (DO_2_) using the formula O_2_ER = VO_2_/DO_2_ = (SaO_2_ − ScvO_2_)/SaO_2_.

### 2.5. Calculation of Mean Circulatory Filling Pressure Analogue and Related Variables

The methods of the Pmca algorithm have been described in detail previously [22,36,37,38]. Briefly, based on a Guytonian model of the systemic circulation (CO = *V*R = (Pmcf − CVP)/R_VR_), an analogue of Pmcf can be derived using the mathematical model Pmca = (*a* × CVP) + (*b* × MAP) + (*c* × CO) [21,39]. In this formula, *a* and *b* are dimensionless constants (*a* + *b* = 1). Assuming a veno-arterial compliance ratio of 24:1, *a* = 0.96 and *b* = 0.04, reflecting the contribution of venous and arterial compartments, and *c* resembles arteriovenous resistance and is based on a formula including age, height, and weight [21]:

$$c=\frac{0.038\;\left(94.17+0.193\times age\right)}{4.5\;\left(0.99^{age-15}\right)\;0.007184\;\cdot \;\left(height^{0.725}\right)\;\left(weight^{0.425}\right)}$$

In addition, the following values were determined: (1) pressure gradient for venous return (PG_VR_) was defined as the pressure difference between Pmca and CVP (PG_VR_ = Pmca − CVP); and (2) resistance to venous return (R_VR_) was defined as the resistance downstream of Pmca to reflect resistance for venous return and was calculated as the ratio of the pressure difference between Pmca and CVP and CO (R_VR_ = (Pmca − CVP)/CO). This formula is used to describe venous return during transient states of imbalances (Pmca is the average pressure in the systemic circulation and R_VR_ is the resistance encountered to the heart) [40,41].

### 2.6. Sublingual Microcirculation Analysis

Sublingual microcirculation was assessed 30 min after induction of general anesthesia, before surgical incision, using SDF+ imaging (Microscan; Microvision Medical BV, Amsterdam, The Netherlands), in accordance with the guidelines on the assessment of sublingual microcirculation of the European Society of Intensive Care Medicine [42]. We recorded sublingual microcirculation videos from at least five sites. To optimize video quality, we tried to avoid pressure and movement artefacts, optimized focus and illumination, and cleaned saliva and/or blood from the sublingual mucosa. Investigators who recorded microcirculation were blinded to systemic hemodynamic variables and vice versa.

Before analysis, all sublingual perfusion videos were evaluated by two experienced raters blinded to all patient data, according to a modified microcirculation image quality score (MIQS) [42,43]. The best three videos from each recording were analyzed offline by a blinded investigator, both manually and with the AVA4.3C Research Software (Microvision Medical, Amsterdam, the Netherlands) [42,44]. We analyzed the De Backer score and De Backer score (small) as density scores, and the Consensus Proportion of Perfused Vessels (Consensus PPV) and Consensus PPV (small) as flow scores. Vessel diameter, vessel length, and RBC velocity were determined with the latest version of AVA software using a modified optical flow-based algorithm. The method uses per video frame data to measure the overall velocity per vessel segment.

### 2.7. Fluid Mechanics and Computational Fluid Dynamics

Computational fluid dynamics is the process of mathematically modelling a physical phenomenon involving fluid flow and numerically solving it using the advances in computational mathematics, numerical analysis, and computers. We performed a numerical study utilizing the CFD approach and randomly selected patient data from the recorded sublingual microcirculation videos. Randomization was achieved by using random computer-generated numbers.

We reconstructed the two-dimensional (2D)-sublingual microvessels and applied CFD to evaluate the correlation of Pmca and RBC velocity with the obtained pressure and velocity fields in microvessels from the CFD approach (i.e., pressure difference (Δp)) under laminar flow assumption. For the blood flow in a vessel, Δp is the pressure difference between any two points along its given length, describing the main driving force of blood motion in the vessel.

Initially, the domain was reconstructed from the 2D images. The reconstructed fluid domain was then discretized in a 2D computational mesh of more than a few thousand quadrilateral elements. The numerical scheme was converged when the residuals (errors) of the momentum and continuity equations were less than or equal to the predetermined error, i.e., residual error = 10^−6^, in this study. At the inlet(s), we considered a constant velocity profile obtained from the measurements of this study, specific for each patient. At the outlet(s), a pressure outlet condition was applied, meaning that the pressure at the outlet had a predefined value. Finally, a no slip condition was applied to the microvessel walls, following the rigid wall assumption [45].

The equations of fluid flow are the continuity and momentum (Navier-Stokes) equations. These equations form a non-linear system of partial differential equations (PDEs). For performing CFD analysis, we assume that blood is an incompressible, Newtonian fluid. The governing equations of motion, the conservation of mass, and the Navier-Stokes equations are written in vector form as follows:

$$\{\begin{array}{c} \nabla \cdot u=0,\\ \frac{\partial u}{\partial t}+\left(u\cdot \nabla \right)u=-\frac{1}{\rho }\nabla p+v\nabla^{2}u, \end{array}$$

where u is the velocity vector, p is the blood pressure, $v=3.2\times 10^{-6}\;m^{3}{\;s}^{-1}$ is the kinematic viscosity, and $\rho =1050\;{\mathrm{kg}\;m}^{-3}$ is the blood density.

**Figure 1:**
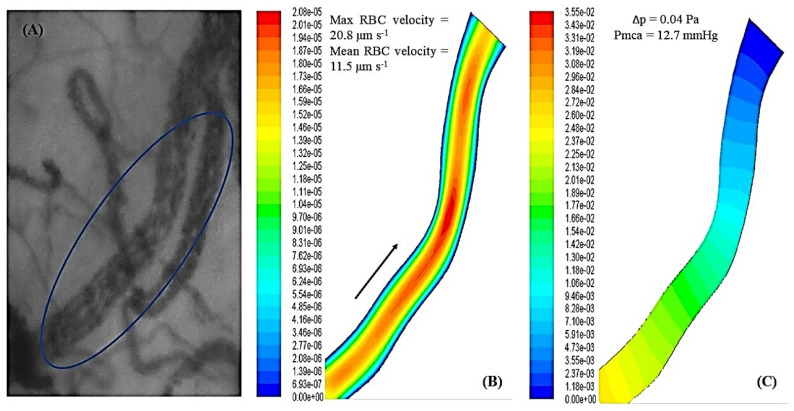
(**A**) is the reconstructed microvessel from a recorded sublingual microcirculation video; (**B**) is the obtained flow field after application of computational fluid dynamics (the arrow shows the flow direction); and (**C**) is the obtained pressure field after application of computational fluid dynamics for the specific microvessel.

The above equations were solved in their discretized form using the finite volume methodology. The discretized algebraic system was solved using the semi-implicit method for pressure linked equation algorithm (SIMPLE) [46]. Steady-state numerical simulations were performed using the finite volume-based software package Ansys Fluent (Ansys Inc., Canonsburg, PA, USA). Figure 1 highlights the CFD approach developed for this study.

### 2.8. Statistical Analysis

The statistical significance of hemodynamic variations between the variables analyzed in each microcirculation video was determined by non-parametric ANOVA tests. The studied variables are presented with their mean value and standard deviation (mean ± SD). The Kendall’s rank correlation between multiple time series was utilized for correlating data [47]. In this test, we conducted a hypothesis test to determine which correlations are significantly different from zero. Due to the study sample (*n* = 20), post-hoc bootstrapping metrics were used to allow estimation of the sampling distribution using random sampling methods. The analysis, including the post-hoc bootstrapping, was performed in Matlab (MathWorks, Natick, MA, USA). *p* values less than 0.05 were deemed significant.

## 3. Results

Twenty patients were included in the study, of whom 12 (60%) were men and 8 (40%) were women, with a median age of 39.5 years (IQR 35.5–44.5). Demographics and clinical characteristics are shown in Table S1, while the anesthetic parameters 30 min after induction of anesthesia are depicted in Table S2.

### 3.1. Baseline Systemic and Sublingual Microcirculation Variables

**Figure 2:**
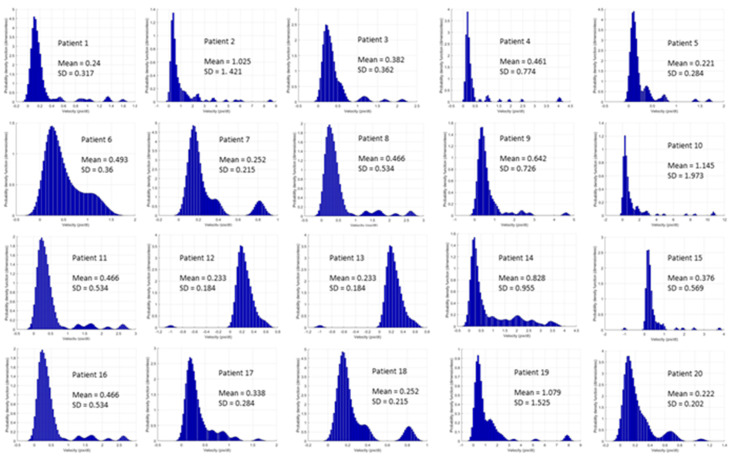
Probability density functions of velocity distributions. Note the velocity values (mean) and the standard deviation (SD). Transverse axis: RBC velocity (pix dt^-1^); Vertical axis: Probability density function (dimensionless).

Baseline hemodynamic and metabolic parameters were within the normal range (Table 1 and Table 2). Mean arterial pressure was maintained ≥65 mmHg without vasopressor administration. Sublingual velocity distributions were similar and followed a log-normal distribution, but distinct differences with different mean values were observed from case to case (Figure 2). An additional statistical analysis with a non-parametric ANOVA test showed that the velocity distributions were significantly different among patients (*p* < 0.001). The aforementioned physiological characteristics were translated into a mean DO_2_ and VO_2_ of 973.8 ± 116.2 mL min^−1^ and 247.4 ± 35.6 mL min^−1^, respectively.

### 3.2. Correlation of Mean Circulatory Filling Pressure Analogue with Systemic and Sublingual Microcirculation Variables

The correlation of Pmca with systemic hemodynamic variables and sublingual microcirculatory flow and density variables is depicted in Table 3.

### 3.3. Correlation of Mean Circulatory Filling Pressure Analogue with Sublingual Red Blood Cell Velocity and Microvessel Length

**Figure 3:**
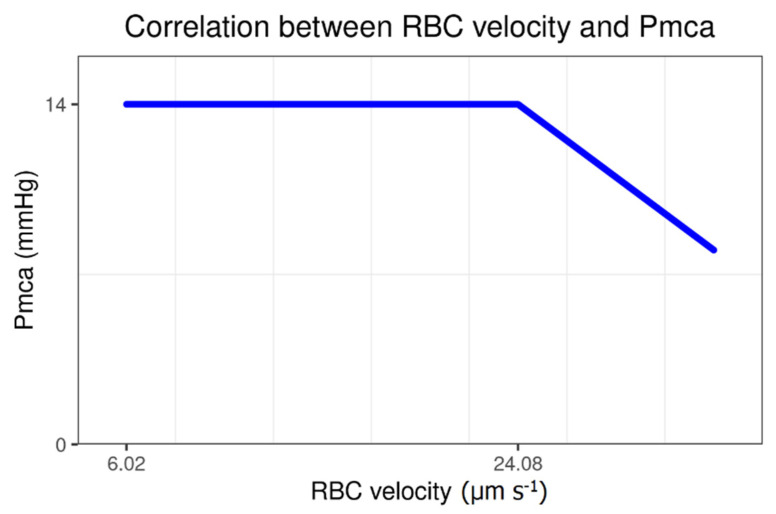
Correlation of Pmca with sublingual microcirculation RBC velocity in individuals with steady-state physiology, effective coupling between the macro- and microcirculation, and normal tissue oxygen extraction ratio. A constant Pmca of 14 mmHg was observed in individuals with RBC velocity 6–24 μm s^−1^, while a Pmca of <14 mmHg was observed in those with RBC velocity > 24 μm s^−1^. Pmca, mean circulatory filling pressure analogue; RBC, red blood cell.

A negative correlation was observed between Pmca and RBC velocity (*r* = −0.03, *p* = 0.87). Interestingly, a constant Pmca value of 14 mmHg was observed in all individuals with sublingual RBC velocity 6–24 μm s^−1^. On the contrary, a Pmca < 14 mmHg was observed in those with RBC velocity > 24 μm s^−1^ (Figure 3). In addition, a positive correlation between Pmca and sublingual microvessel length was observed (*r* = 0.04, *p* = 0.82; Figure S1).

### 3.4. Correlation of Sublingual Red Blood Cell Velocity with Microvessel Diameter and Length

A negative correlation between RBC velocity and microvessel length (*r* = −0.19, *p* = 0.27) was observed in all patients. Additionally, a positive correlation was observed between RBC velocity and microvessel diameter (*r* = 0.68, *p* < 0.001; Figure S2).

### 3.5. Correlation of Sublingual Red Blood Cell Velocity with Sublingual Density and Flow Variables and Oxygen Extraction Ratio

A positive correlation was observed between RBC velocity and De Backer score (*r* = 0.2, *p* = 0.24), De Backer score (small) (*r* = 0.26, *p* = 0.125), and consensus PPV (small) (*r* = 0.07, *p* = 0.73) (Table S3, Figure S3). Furthermore, a positive correlation was observed between RBC velocity and O_2_ER (*r* = 0.034, *p* = 0.87).

### 3.6. Fluid Mechanics and Computational Fluid Dynamics

**Figure 4:**
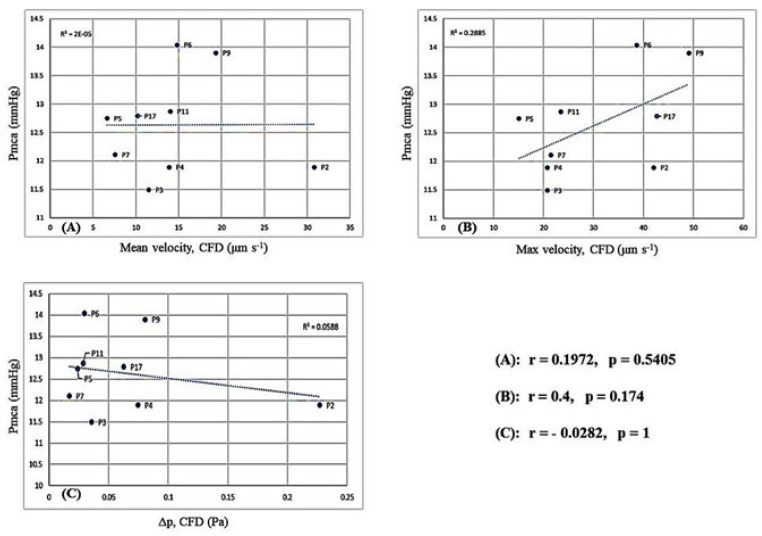
Computational fluid dynamics simulation: (**A**) correlation between Pmca and mean RBC velocity; (**B**) correlation between Pmca and maximum RBC velocity in the domain; and (**C**) correlation between Pmca and pressure difference (Δp).

Reconstruction of the 2D microvessels and the application of CFD to evaluate the velocity and pressure fields in microvessels are depicted in Figures S4–S12. Correlation of Pmca with RBC velocity and Δp is depicted in Figure 4. Interestingly, when Pmca ranged between 11 mmHg and 15 mmHg, Δp fluctuated between 0.02 Pa and 0.1 Pa. Bootstrapping metrics (*n* = 30) revealed a statistically significant negative correlation between Pmca and Δp (*r* = −0.30, *p* = 0.02; Figure S13).

## 4. Discussion

In this explorative study with anesthetized individuals in steady-state physiology, a constant Pmca value of 14 mmHg was observed in those with sublingual RBC velocity 6–24 μm s^−1^, while a Pmca <14 mmHg was observed in individuals with RBC velocity > 24 μm s^−1^. A positive correlation was observed between RBC velocity and O_2_ER. In addition, CFD modeling simulation revealed a negative correlation between Pmca και Δp. When Pmca ranged between 11 mmHg and 15 mmHg, Δp constantly fluctuated between 0.02 Pa and 0.1 Pa. The present study provides a novel understanding of hemodynamic coherence, which can aid in the identification of new hemodynamic phenotypes and microcirculation-guided therapeutic strategies.

An intact coherence between the macro- and microcirculation facilitates DO_2_ to the parenchymal cells. Patients undergoing general anesthesia and critically ill patients may experience a transient or prolonged loss of this coherence, which may lead to tissue hypoperfusion and organ injury. Although the expansion of hemodynamic monitoring to include monitoring of the microcirculation can be helpful in guiding management [42,48], its visualization and assessment remains technically challenging. Therefore, identifying potential systemic hemodynamic variables that would enable prediction of microcirculatory behavior is of utmost interest [49]. In this clinical study with CFD analysis, we report that a Pmca of 14 mmHg is associated with physiological hemodynamic coherence, effective microcirculatory perfusion, and normal oxygen transport to tissue in anesthetized individuals in steady-state physiology. Considering that systemic hemodynamic reference values or thresholds to define microcirculatory alterations as persisting are not available, Pmca may prove an indirect assessment method of tissue perfusion, allowing microcirculation-guided resuscitation and aiding in the identification of novel hemodynamic phenotypes [20,42].

In normal conditions, organ perfusion is dependent upon CO and the vascular resistance across an organ, while in circulatory shock, fluid resuscitation and/or vasopressors are often necessary to achieve maximal tissue RBC perfusion [50]. The present study included individuals with steady-state physiology, intact vascular regulation, and effective coupling between the macro- and microcirculation, which allowed the description of the physiological functional state. Of note, the association between systemic hemodynamic and microcirculation variables may still exist when hemodynamic coherence is lost [49]. This is important for the monitoring and treatment of circulatory abnormalities, especially in the early phase of diseases during which hemodynamic coherence is usually maintained [16,49]. In our study with patients in a dynamic hemodynamic equilibrium, a Pmca value < 14 mmHg was correlated with higher RBC velocity, probably due to an increase in vascular capacitance and sublingual vessel diameter and/or an increase in heart efficiency. However, there must always be a limit under which decreases in Pmca and venous return impair microcirculatory blood flow [11,12,16,17,18,20,51]. On the contrary, a higher Pmca (> 14 mmHg) may be the result of excessive vasoconstriction, eventually resulting in hemodynamic incoherence and tissue hypoperfusion. These can also explain the detrimental effects of higher doses of vasoconstrictive agents and support the recent trend towards a perfusion-centered resuscitation strategy instead of standard pressure-guided treatment [16,17,52,53].

In the present study, mean RBC velocity was 15 ± 9 μm s^−1^, which is significantly lower compared to that reported in other studies including healthy individuals. Edul et al. reported a normal RBC velocity of 1331 ± 90 μm s^−1^ [26,27], while Rovas et al. recently reported a normal RBC velocity of approximately 102 μm s^−1^ [54]. The lower RBC velocity in our anesthetized patients is suggestive of a hypodynamic microcirculatory state, with the O_2_ER indicating a normal balance between DO_2_ and VO_2_. This, together with the microcirculatory density and flow scores in the present study, strengthen the evidence revealing that tissue oxygenation is maintained at very low RBC velocities. Presumably, a low RBC velocity facilitates oxygen transport to tissue, while tissue hypoxia may develop when the velocity of RBCs increases and, thus, their capillary transit time is not sufficient to unload oxygen completely [24,26]. Nevertheless, whether hyperdynamic microcirculatory flow is always associated with tissue hypoxia remains controversial and, currently, the available evidence is not sufficient to rule out different RBC velocities in healthy people [55]. In septic patients, individual changes in sublingual RBC velocity have been correlated with those in CI after a fluid bolus, but in the face of an unchanged perfused vascular density [27]. In others, a fluid challenge may improve O_2_ER by increasing Pmcf and venous return [56]. More translational research is required to develop a deeper understanding of the relationships reported in the present study.

The application of CFD simulations revealed a negative correlation between Pmca and Δp. For the blood flow in a vessel or organ, Δp is the pressure difference between any two points along a given length of the vessel or the difference between the arterial and venous pressures of the organ, respectively. In our CFD analysis with laminar flow conditions, with the vascular resistance being independent of flow and pressure, an increase in resistance would decrease flow at any given Δp. In clinical practice, fluid challenges and vasopressor administration increase the stressed volume (and thus Pmca and CO) until a certain point, but may not always improve microcirculatory perfusion. On the other hand, fluid overload increases CVP, which decreases venous return and retrogradely increases post-capillary venular pressure, thus impairing microcirculatory perfusion [57,58], especially in patients receiving high-dose vasopressor therapy. The association between the Pmca and Δp ranges in our study further enhances the potential of Pmca to serve as a hemodynamic coherence monitoring tool. Bedside estimation of Pmca can track the effective circulating volume [12,16,18,59,60], while a Pmca value of 14 mmHg may indicate an adequate balance between the macro- and microcirculatory perfusion. The post-hoc bootstrapping metrics in the present study strongly encourage the evaluation of our findings in larger studies.

To the best of our knowledge, this is the first report of the association of Pmcf/Pmca with sublingual RBC velocity and Δp. A strength of the study is that data collection and analyses were performed by blinded investigators, thus preventing inter-observer bias and increasing the credibility of study conclusions. Although the present study has a small sample size, bootstrapping metrics revealed a statistically significant negative correlation between Pmca and Δp. Mean age in our patients was 39.5 years and the results of the present analysis may be different in older individuals. In addition, anesthesia can lower resting metabolic rate and reduce global VO_2_ and has been associated with a reduction in the ability of tissue to extract oxygen. In the present study, however, we used desflurane for maintenance because it produces mild and stable effects on the microcirculation compared to other agents [30].

## 5. Conclusions

In anesthetized individuals in steady-state physiology, the intact regulatory mechanisms maintain an effective coupling between systemic hemodynamics, sublingual microcirculatory perfusion, and tissue oxygenation when Pmca is 14 mmHg. Mean circulatory filling pressure analogue could serve as a marker of hemodynamic coherence. New CFD models were developed, based on finite element methods and differential equations, that can be used in future research on hemodynamic coherence in health and disease.

## Figures and Tables

**Table 1:** Baseline systemic hemodynamic and metabolic variables.

Heart rate (bmp)	67.5 ± 7
Systolic arterial pressure (mmHg)	120 ± 7.4
Diastolic arterial pressure (mmHg)	71.3 ± 7.4
Mean arterial pressure (mmHg)	88.1 ± 7
Cardiac output (L min^−1^)	4.8 ± 1
Cardiac index (L min^−1^ m^−2^)	2.6 ± 0.3
Stroke volume (mL beat^−1^)	74.7 ± 9.6
Stroke volume variation (%)	5.9 ± 1.8
Systemic vascular resistance (dynes sec cm^−5^)	1306.3 ± 176.3
Central venous pressure (mmHg)	7.1 ± 0.7
Analogue of mean circulatory filling pressure (mmHg)	13.1 ± 0.9
Pressure gradient for venous return (mmHg)	5.9 ± 0.8
Resistance to venous return (mmHg min^−1^ L^−1^)	1.2 ± 0.2
Oxygen delivery (mL min^−1^)	973.8 ± 116.2
Oxygen consumption (mL min^−1^)	247.4 ± 35.6
Oxygen extraction ratio (%)	25.8 ± 2.3
Fraction of inspired oxygen (%)	0.3 ± 0.03
pH	7.39 ± 0.02
PaO_2_ (mmHg)	92.5 ± 5.1
PaCO_2_ (mmHg)	39.2 ± 1.3
HCO_3_ (mmol L^−1^)	25.6 ± 1
Base deficit (mmol L^−1^)	2.08 ± 0.2
Hemoglobin (g dL^−1^)	14.1 ± 0.94
Glucose (mg dL^−1^)	113.6 ± 6.2
Lactate (mmol L^−1^)	0.8 ± 0.2
SpO_2_ (%)	99.6 ± 0.5
SaO_2_ (%)	100 ± 0.0
ScvO_2_ (%)	74.2 ± 2.3
v-aPCO_2_ (mmHg)	2.8 ± 0.9

Data presented as mean ± SD. PaO_2_, arterial partial pressure of oxygen; PaCO_2_, arterial partial pressure of carbon dioxide; SpO_2_, oxygen saturation of hemoglobin; SaO_2_, arterial oxygen saturation; ScvO_2_, central venous oxygen saturation; v-aPCO_2_, venous-to-arterial carbon dioxide difference.

**Table 2:** Baseline sublingual microcirculation variables.

De Backer score (mm^−1^)	3.7 ± 1.2
De Backer score (small) (mm^−1^)	2 ± 1.1
Consensus PPV (%)	94.2 ± 5.7
Consensus PPV (small) (%)	88.2 ± 10
Vessel length (μm)	137.3 ± 96.8
Vessel diameter (μm)	17.2 ± 4
Velocity of red blood cells (μm s^−1^)	15 ± 9

Data presented as mean ± SD. PPV, proportion of perfused vessels.

**Table 3:** Correlation of Pmca with systemic and sublingual microcirculation variables.

**Systemic Hemodynamic Variables**	**Spearman’s rho**	***p*-Value**
Cardiac output (L min^−1^)	0.173	0.31
Cardiac index (L min^−1^ m^−2^)	0.145	0.41
Mean arterial pressure (mmHg)	0.398	0.012
Systemic vascular resistance (dynes s cm^−5^)	0.058	0.75
Pressure gradient for venous return (mmHg)	0.438	0.008
Resistance of venous return (mmHg min^−1^ L^−1^)	0.203	0.23
**Sublingual Microcirculation Variables**	**Spearman’s rho**	***p*-Value**
De Backer score (mm^−1^)	−0.189	0.27
De Backer score (small) (mm^−1^)	0.011	0.97
Consensus PPV (%)	0.017	0.95
Consensus PPV (small) (%)	0.06	0.74

PPV, proportion of perfused vessels.

## Data Availability

Data can be made available upon request after publication through a collaborative process. Researchers should provide a methodically sound proposal with specific objectives in an approval proposal. Please contact the corresponding author for additional information.

## Supplementary Materials

The following supporting information can be downloaded at: https://www.mdpi.com/article/10.3390/jcm11164885/s1, Figure S1: Correlations between Pmca, microvessel length, and RBC velocity; Figure S2. Correlations of RBC velocity with microvessel length and diameter; Figure S3. Correlation of sublingual RBC velocity with consensus PPV (small), De Backer score (small), and De Backer score; Figure S4. Reconstruction of the 2D microvessel and application of CFD to evaluate the velocity and pressure fields in microvessels; Figure S5. Reconstruction of the 2D microvessel and application of CFD to evaluate the velocity and pressure fields in microvessels; Figure S6. Reconstruction of the 2D microvessel and application of CFD to evaluate the velocity and pressure fields in microvessels; Figure S7. Reconstruction of the 2D microvessel and application of CFD to evaluate the velocity and pressure fields in microvessels; Figure S8. Reconstruction of the 2D microvessel and application of CFD to evaluate the velocity and pressure fields in microvessels; Figure S9. Reconstruction of the 2D microvessel and application of CFD to evaluate the velocity and pressure fields in microvessels; Figure S10. Reconstruction of the 2D microvessel and application of CFD to evaluate the velocity and pressure fields in microvessels; Figure S11. Reconstruction of the 2D microvessel and application of CFD to evaluate the velocity and pressure fields in microvessels; Figure S12. Reconstruction of the 2D microvessel and application of CFD to evaluate the velocity and pressure fields in microvessels; Figure S13. Bootstrapping metrics analysis (*n* = 30) investigating the correlation between Pmca and Δp; Table S1. Baseline characteristics of the patients; Table S2. Baseline anesthetic parameters 30 min after induction of anesthesia (mean ± SD); Table S3. Correlation of sublingual RBC velocity with sublingual density and flow variables.
