# Supplementary material for: Relationship of Effective Circulating Volume with Sublingual Red Blood Cell Velocity and Microvessel Pressure Difference: A Clinical Investigation and Computational Fluid Dynamics Modeling
Source: J Clin Med. 2022 Aug 20;11(16):4885. doi: 10.3390/jcm11164885 (PMC9410298; doi:10.3390/jcm11164885)
Supplement: Supplementary file 1 [file jcm-11-04885-s001.zip › jcm-1836896-supplementary.pdf]

Supplemental Material

Figure S1

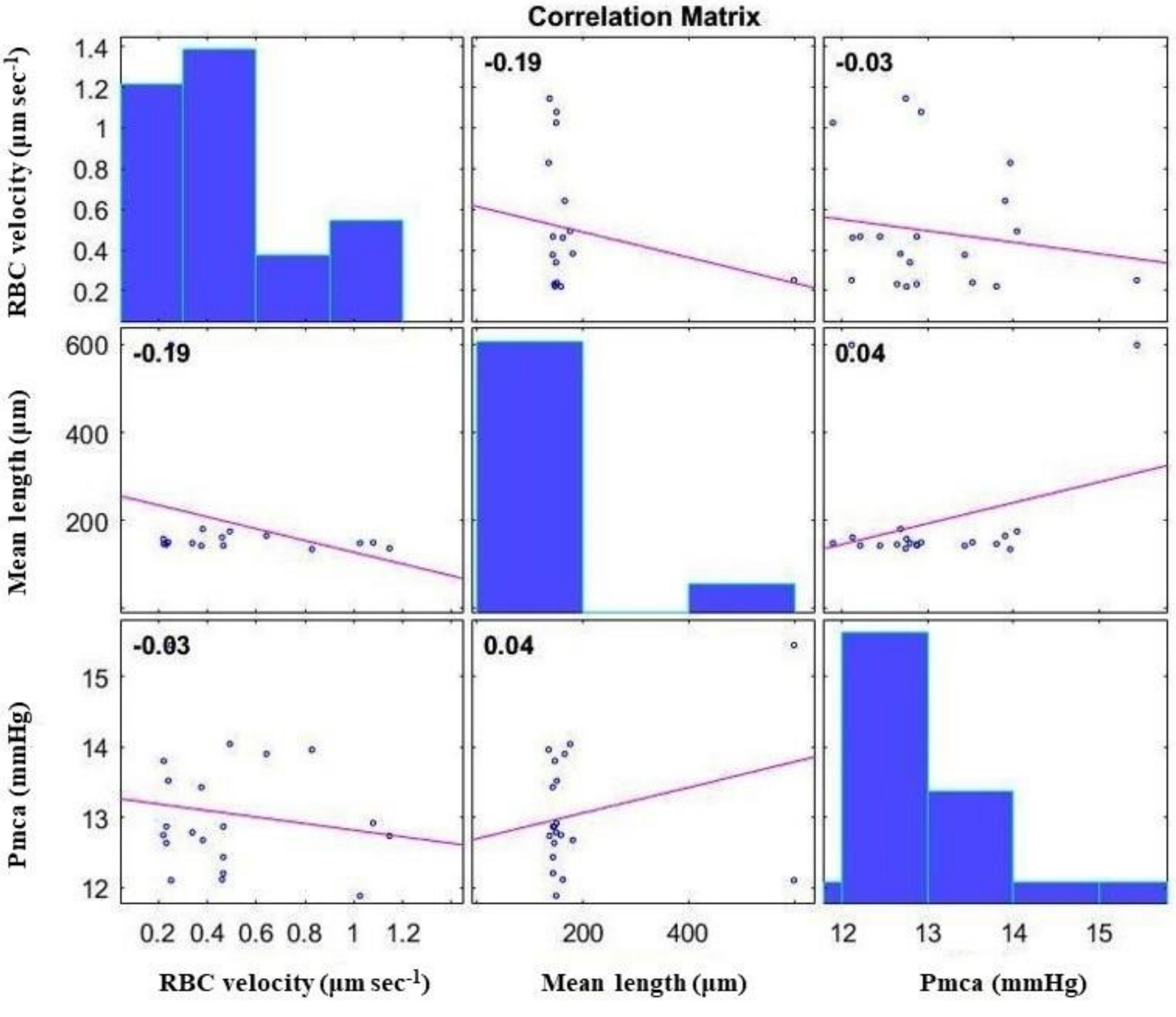

Figure S1. Correlations between Pmca, microvessel length, and RBC velocity.

Figure S2

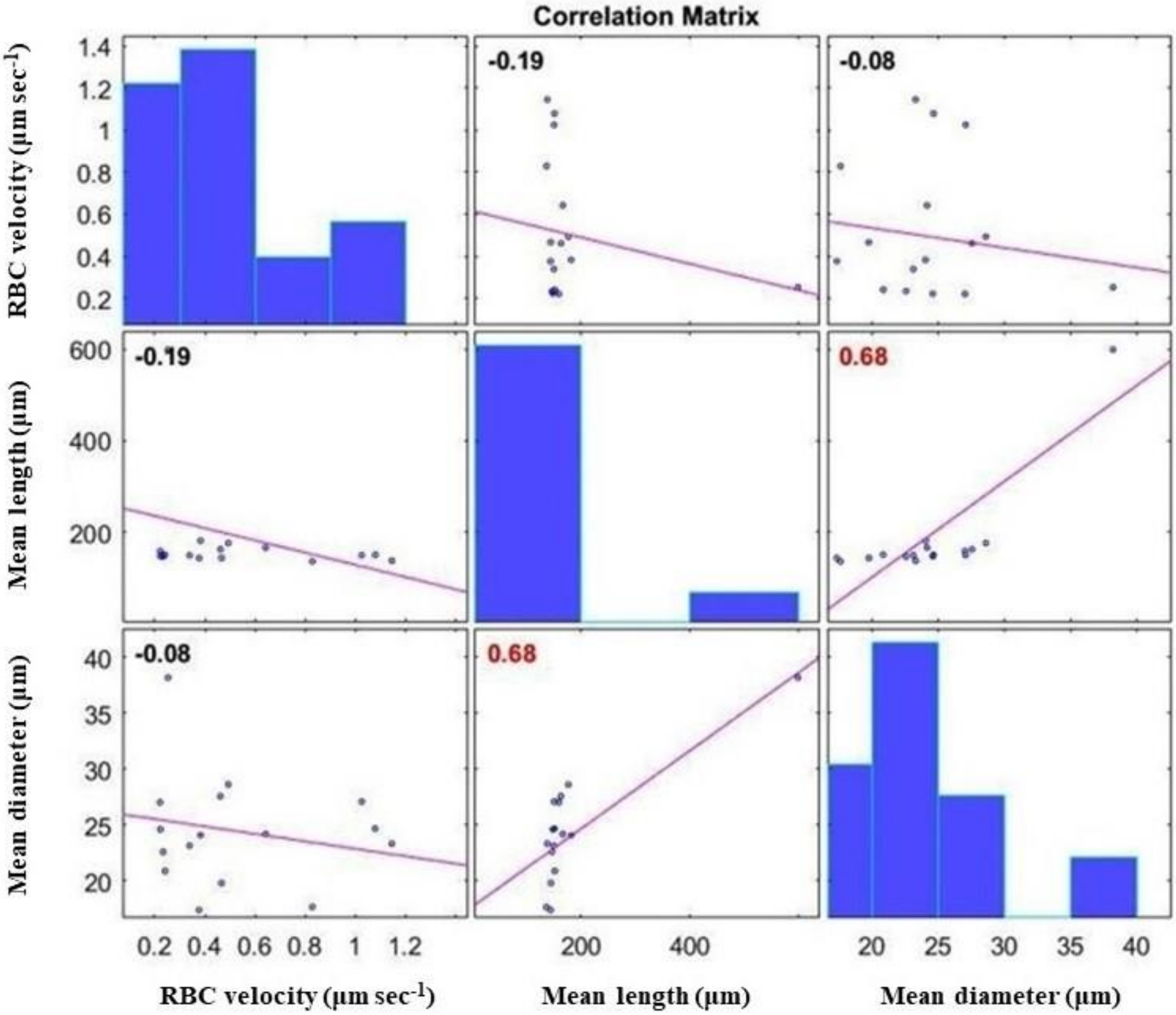

Figure S2. Correlations of RBC velocity with microvessel length and diameter.

Figure S3

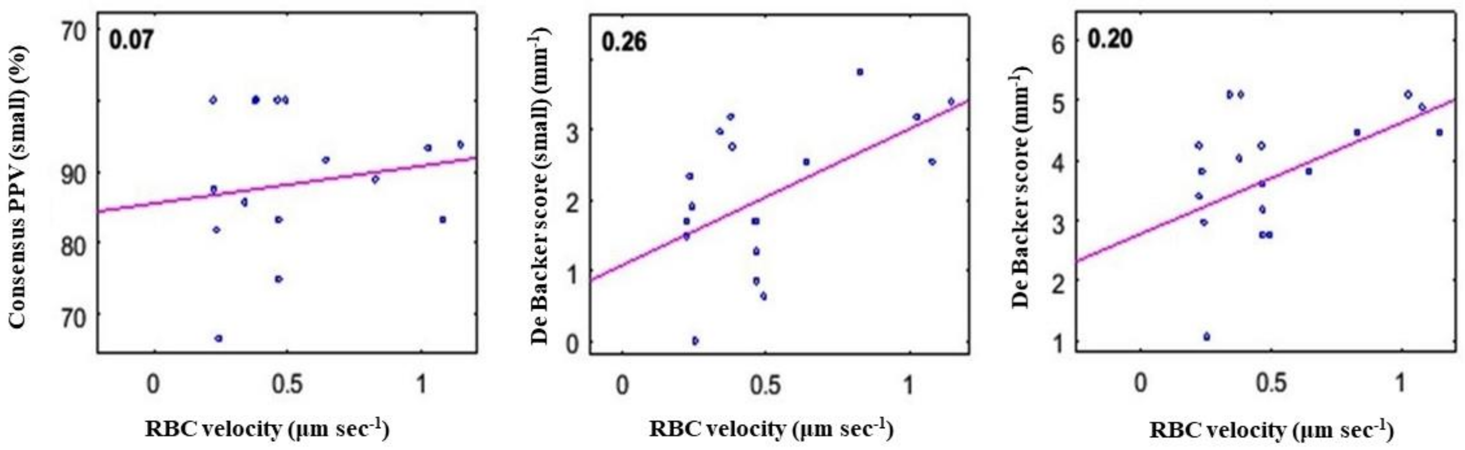

Figure S3. Correlation of sublingual RBC velocity with Consensus PPV (small), De Backer score (small), and De Backer score.

Figure S4

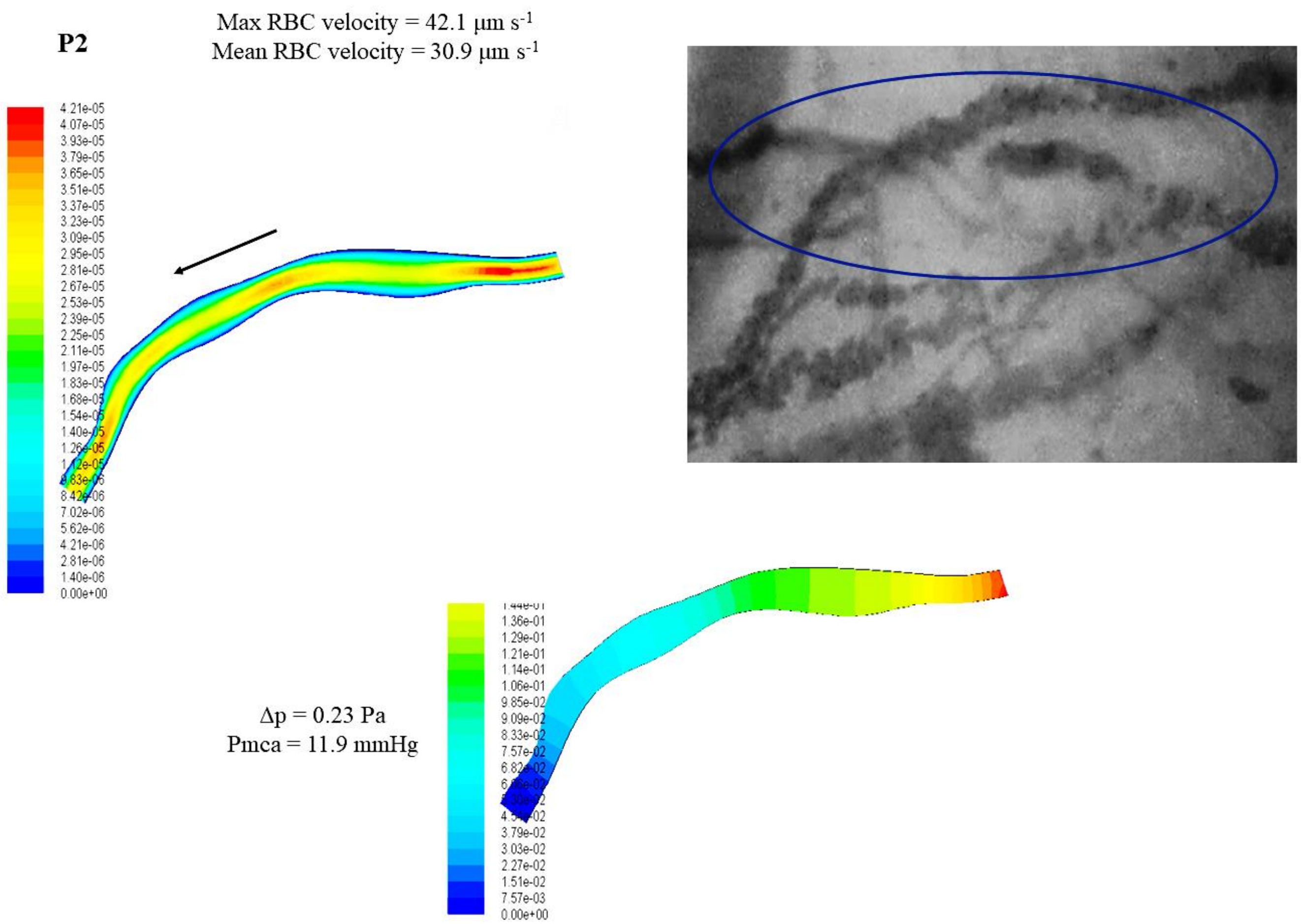

Figure S4. Reconstruction of the 2D microvessel and application of CFD to evaluate the velocity and pressure fields in microvessels.

Figure S5

P3

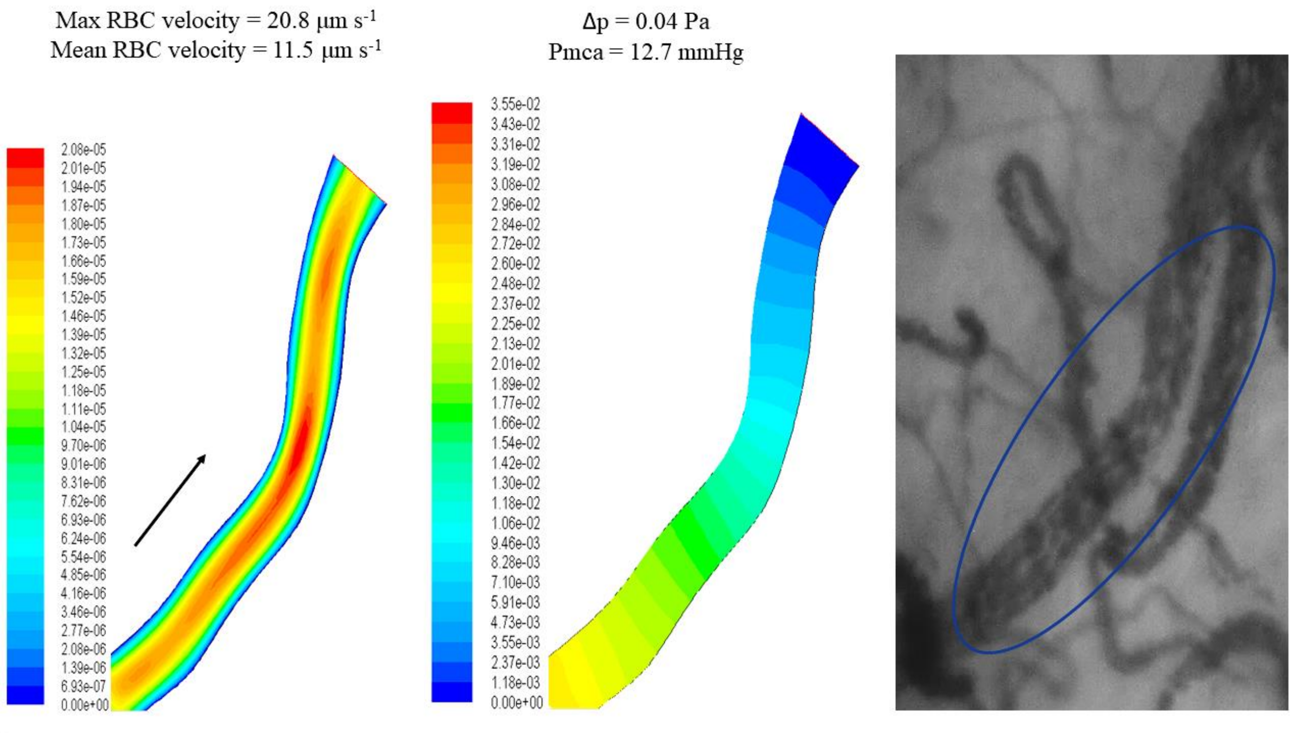

Figure S5. Reconstruction of the 2D microvessel and application of CFD to evaluate the velocity and pressure fields in microvessels.

Figure S6

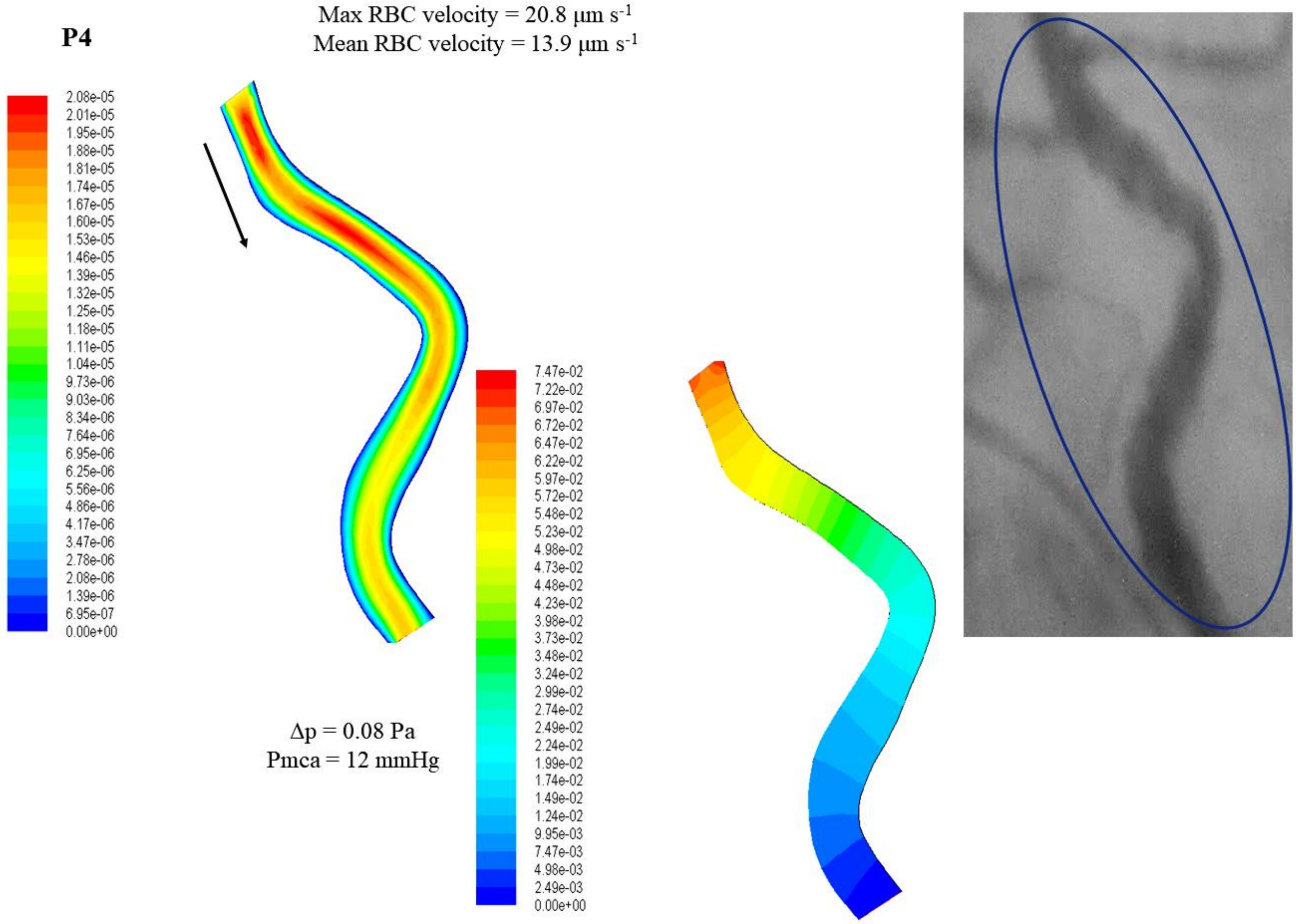

Figure S6. Reconstruction of the 2D microvessel and application of CFD to evaluate the velocity and pressure fields in microvessels.

Figure S7

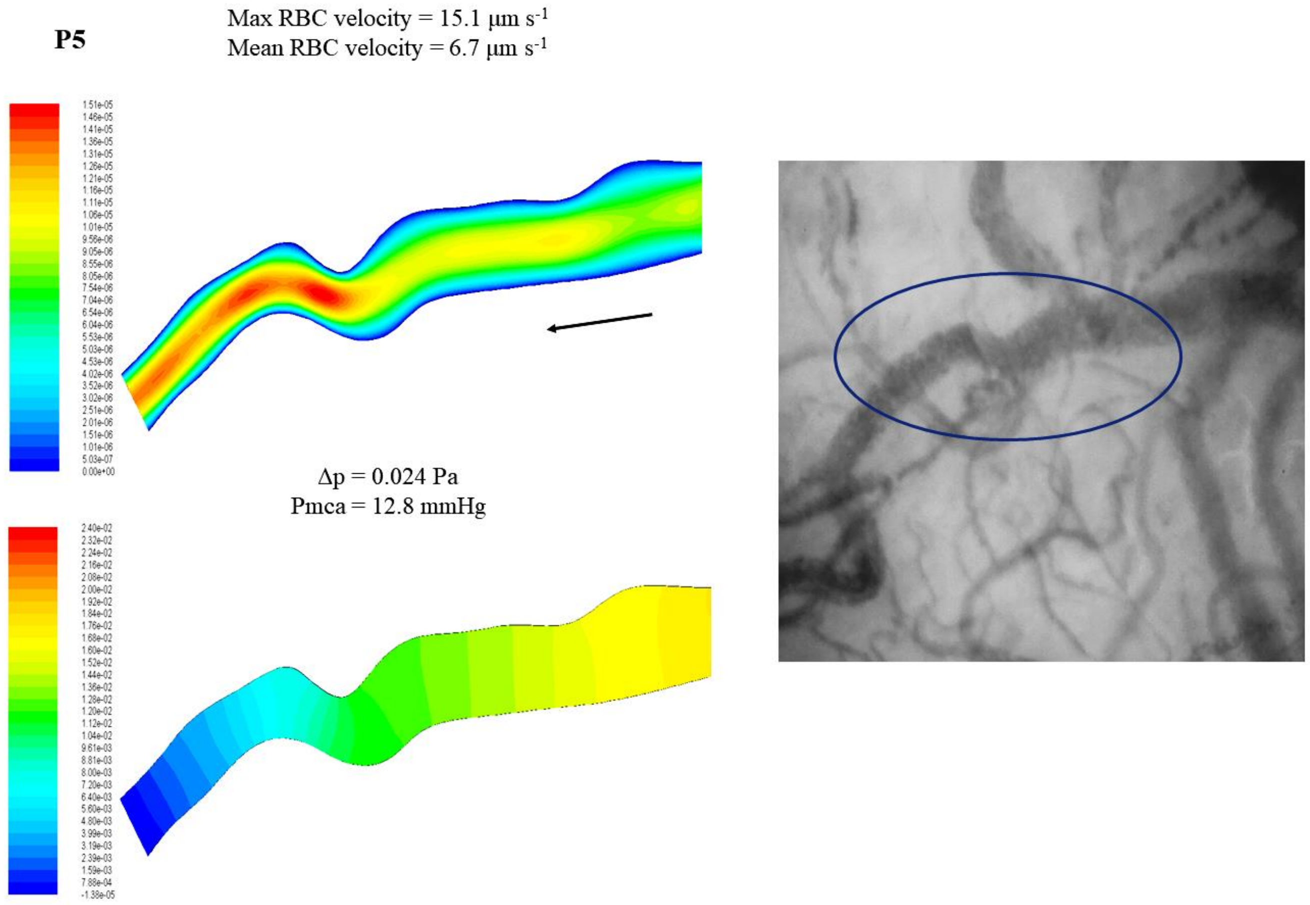

Figure S7. Reconstruction of the 2D microvessel and application of CFD to evaluate the velocity and pressure fields in microvessels.

Figure S8

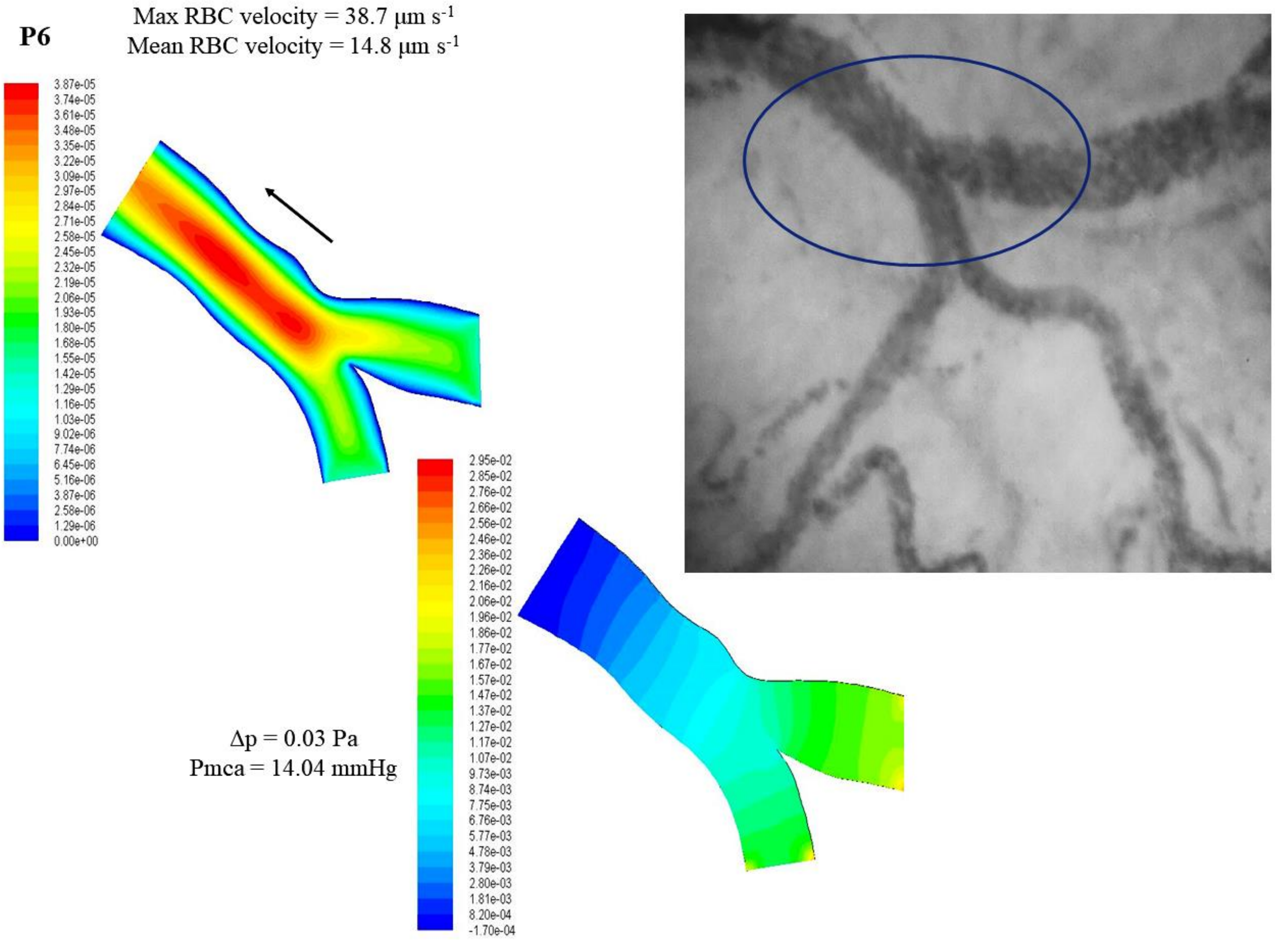

Figure S8. Reconstruction of the 2D microvessel and application of CFD to evaluate the velocity and pressure fields in microvessels.

Figure S9

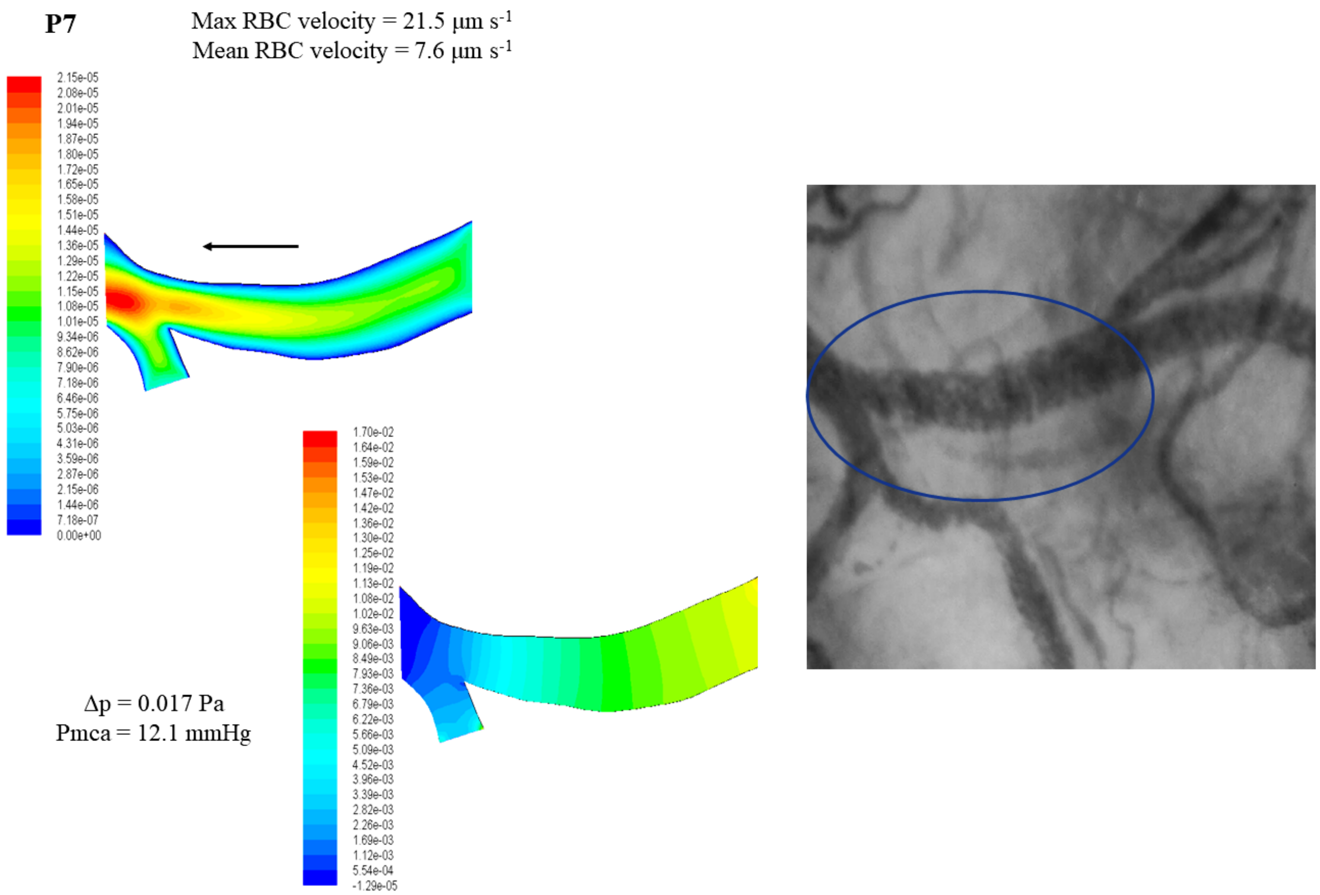

Figure S9. Reconstruction of the 2D microvessel and application of CFD to evaluate the velocity and pressure fields in microvessels.

Figure S10

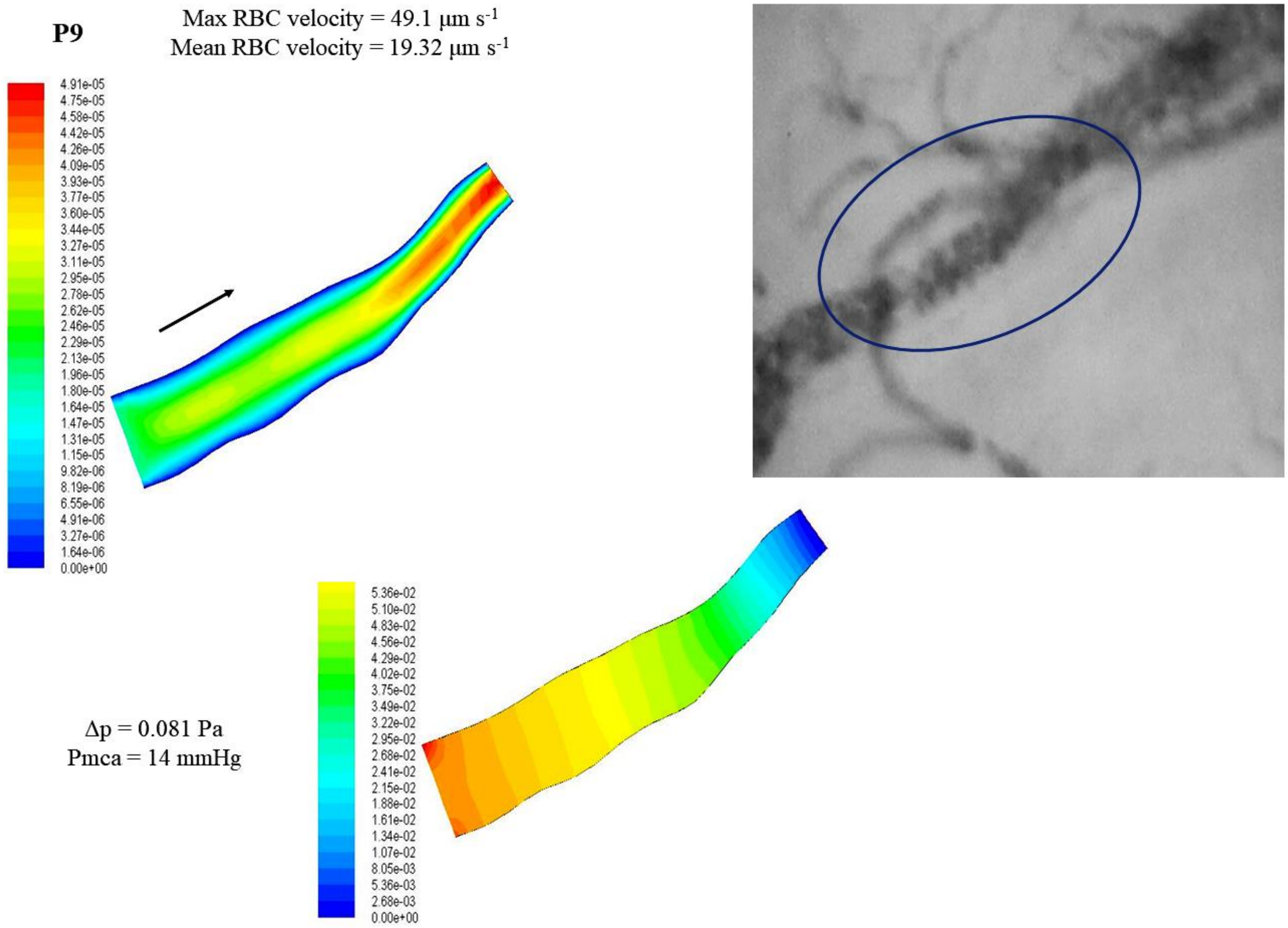

Figure S10. Reconstruction of the 2D microvessel and application of CFD to evaluate the velocity and pressure fields in microvessels.

Figure S11

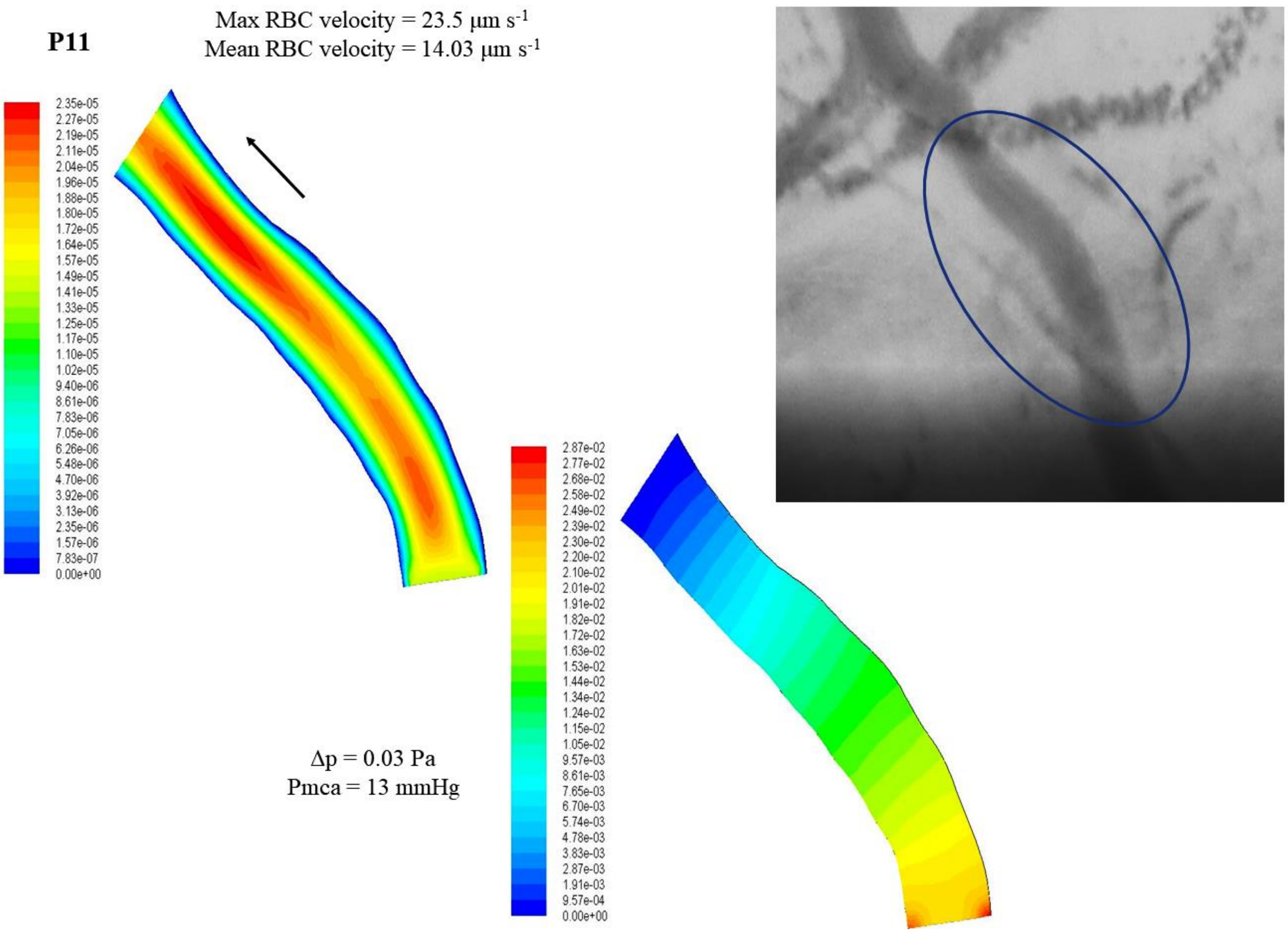

Figure S11. Reconstruction of the 2D microvessel and application of CFD to evaluate the velocity and pressure fields in microvessels.

Figure S12

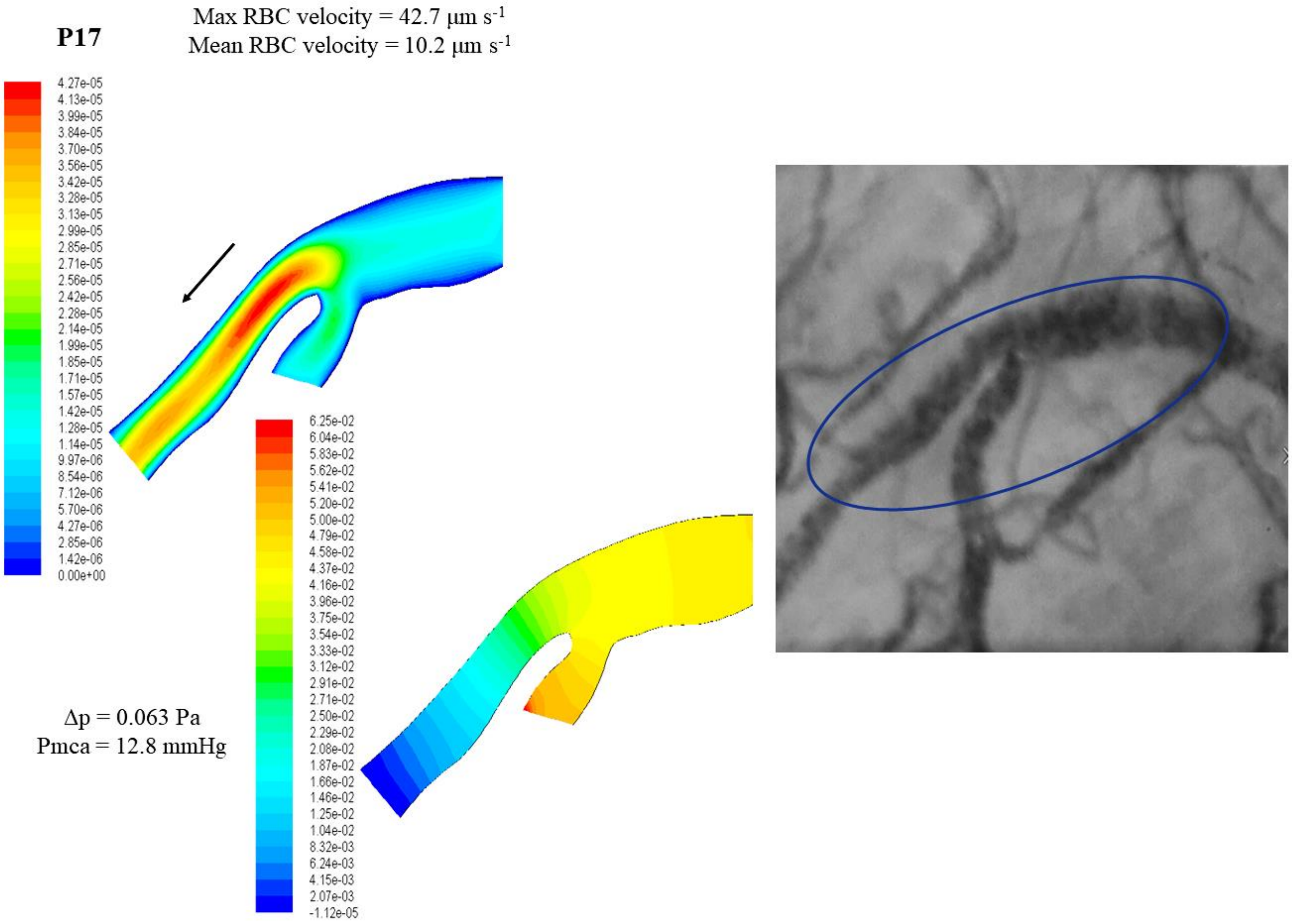

Figure S12. Reconstruction of the 2D microvessel and application of CFD to evaluate the velocity and pressure fields in microvessels.

Figure S13

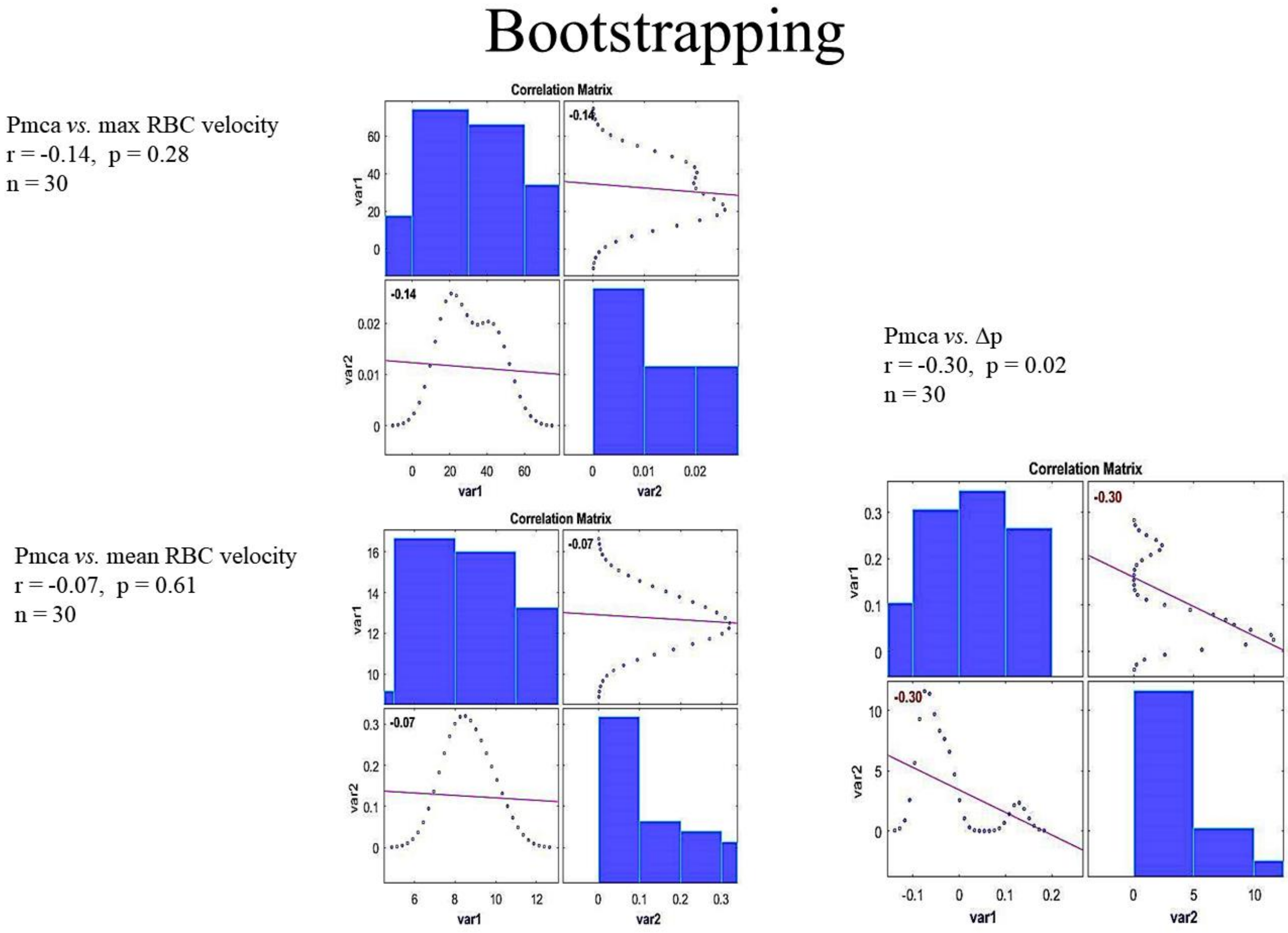

Figure S13. Bootstrapping metrics analysis (n=30) investigating the correlation between Pmca and Δp.

Table S1. Baseline characteristics of the patients

Age (years), median (IQR)	39.5 (35.5-44.5)
Sex - male, n (%)	12 (60)
Sex - female, n (%)	8 (40)
BMI (kg m^{-2}), median (IQR)	25.1 (23.15-25.8)
Body Surface Area (m^2), median (IQR)	1.94 (1.83-2.02)
Type of planned surgery	
Gastrointestinal, n (%)	7 (35)
Gynecological, n (%)	3 (15)
Thyroidectomy, n (%)	2 (10)
Urological, n (%)	8 (40)

Table S2. Baseline anesthetic parameters 30 min after induction of anesthesia (mean ± SD)

Bispectral index	42.5 ± 1.5
Temperature (°C)	36.7 ± 0.1
Tidal volume (ml kg^{-1} IBW)	560 ± 52.2
Respiratory rate (min^{-1})	13.7 ± 1.1
Positive end-expiratory pressure (cmH_2O)	5 ± 0
End-tidal carbon dioxide (mmHg)	36.9 ± 0.9
Peak inspiratory pressure (cmH_2O)	17.8 ± 1.3
Plateau pressure (cmH_2O)	16 ± 1.4

IBW, ideal body weight.

Table S3. Correlation of sublingual RBC velocity with sublingual density and flow variables

	RBC velocity	Consensus PPV (small)	Consensus PPV	De Backer score (small)	De Backer score
RBC velocity		**0.07**	-0.02	**0.26**	**0.2**
Consensus PPV (small)	**0.07**		**0.84**	**0.19**	**0.32**
Consensus PPV	-0.02	**0.84**		-0.13	**0.04**
De Backer score (small)	**0.26**	**0.19**	-0.13		**0.68**
De Backer score	**0.2**	**0.32**	**0.04**	**0.68**	

Red color: statistically significant positive correlations; Black bold: positive correlations.
